# Attack of the clones: Population genetics reveals clonality of *Colletotrichum lupini*, the causal agent of lupin anthracnose

**DOI:** 10.1111/mpp.13332

**Published:** 2023-04-20

**Authors:** Joris A. Alkemade, Riccardo Baroncelli, Monika M. Messmer, Pierre Hohmann

**Affiliations:** ^1^ Department of Crop Sciences Research Institute of Organic Agriculture (FiBL) Frick Switzerland; ^2^ Department of Agricultural and Food Sciences (DISTAL) University of Bologna Bologna Italy; ^3^ Centre for Studies on Bioinspired Agro‐Enviromental Technology, Università di Napoli Federico II Portici 80055 Italy; ^4^ Bonaplanta, BioCrops Innovations SL Manresa Spain

**Keywords:** anthracnose, *Colletotrichum*, lupin, population genetics, RAD sequencing

## Abstract

*Colletotrichum lupini*, the causative agent of lupin anthracnose, affects lupin cultivation worldwide. Understanding its population structure and evolutionary potential is crucial to design successful disease management strategies. The objective of this study was to employ population genetics to investigate the diversity, evolutionary dynamics, and molecular basis of the interaction of this notorious lupin pathogen with its host. A collection of globally representative *C. lupini* isolates was genotyped through triple digest restriction site‐associated DNA sequencing, resulting in a data set of unparalleled resolution. Phylogenetic and structural analysis could distinguish four independent lineages (I–IV). The strong population structure and high overall standardized index of association (*r̅*_d_) indicates that *C. lupini* reproduces clonally. Different morphologies and virulence patterns on white lupin (*Lupinus albus*) and Andean lupin (*Lupinus mutabilis*) were observed between and within clonal lineages. Isolates belonging to lineage II were shown to have a minichromosome that was also partly present in lineage III and IV, but not in lineage I isolates. Variation in the presence of this minichromosome could imply a role in host–pathogen interaction. All four lineages were present in the South American Andes region, which is suggested to be the centre of origin of this species. Only members of lineage II have been found outside South America since the 1990s, indicating it as the current pandemic population. As a seedborne pathogen, *C. lupini* has mainly spread through infected but symptomless seeds, stressing the importance of phytosanitary measures to prevent future outbreaks of strains that are yet confined to South America.

## Graphical abstract

Population genetics of *Colletotrichum lupini* revealed four distinct lineages, of which one is globally spread, indicated clonal reproduction, and showed variability in the presence/absence of a minichromosome.

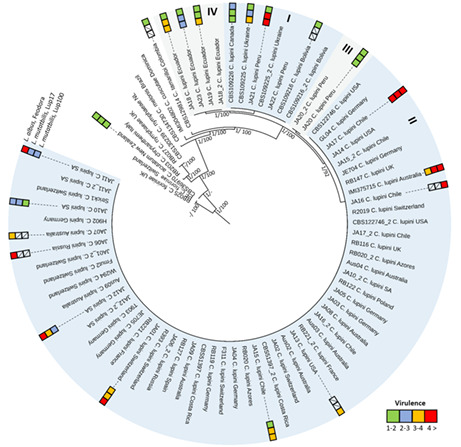

## 1 INTRODUCTION

Fungal pathogens cause severe crop losses worldwide. Current agricultural practices, the development of fungicide‐resistant pathogens, the increase of global trade and movement, loss of biodiversity, and a warming climate likely tend to shift the pathogen distribution and increase disease pressure and outbreak frequency (Corredor‐Moreno & Saunders, 2020; Fones et al., 2020). Policy shifts towards the phasing out of fungicides and the aim for a more sustainable agriculture require a better understanding of pathogen population dynamics to design adequate breeding and disease management strategies. Population genetics offers great potential to get insight in the evolutionary processes that affect genetic diversity and population structure of fungal plant pathogens (McDonald & Linde, 2002). In many important fungal and oomycete plant pathogens, such as *Peronospora destructor* on onion (Van der Heyden et al., 2022), *Fusarium oxysporum* on cotton (Halpern et al., 2020), and *Colletotrichum graminicola* on maize (Rogério et al., 2023), population genetics provided essential information on pathogen evolution and diversity. Knowledge on pathogen population structure is crucial for developing long‐term disease management strategies as was pointed out by Wallace et al. (2020) for *Pseudoperonospora cubensis* on Cucurbitaceae.

*Colletotrichum* species are listed in the top 10 of most important fungal plant pathogens and cause major crop losses worldwide (Dean et al., 2012). Over 200 *Colletotrichum* species, which are divided among 15 species complexes, are currently described (Talhinhas & Baroncelli, 2021). Species belonging to the *Colletotrichum acutatum* species complex are especially notorious, with members causing pre‐ and postharvest disease in fruit, cereal, and legume crops (Bragança et al., 2016; Damm et al., 2012). Devastating economic losses are often observed for strawberry (Baroncelli et al., 2015), citrus (Guarnaccia et al., 2017), and olive (Moral & Trapero, 2009). Acute ends of its conidia are the most characteristic of the species complex (Damm et al., 2012), but differentiation purely based on morphology has proven to be extremely hard (Cannon et al., 2000). Hemibiotrophy is the most common lifestyle within the complex, but purely biotrophic, necrotrophic, and endophytic lifestyles have been observed as well (De Silva et al., 2017; Peres et al., 2005). Besides being devastating plant pathogens, members of the *C. acutatum* species complex offer great potential to serve as model organisms to study host–pathogen evolution (Baroncelli et al., 2017).

*Colletotrichum lupini*, a Clade 1 member of the *C. acutatum* species complex, causes anthracnose, which is the most devastating lupin disease worldwide (Damm et al., 2012; Talhinhas et al., 2016). The disease is seed‐ and airborne with typical symptoms being stem twisting and necrotic lesions on stems and pods (Alkemade, Messmer, Voegele, et al., 2021). The most agriculturally important lupin species are blue lupin (*Lupinus angustifolius*), white lupin (*Lupinus albus*), and Andean lupin (*Lupinus mutabilis*) (Wolko et al., 2011). Andean lupin plays a major role in regional food security, while blue and white lupin mostly serve as feed for the livestock industry. Most lupin production takes currently place in Western Australia (47%; FAOSTAT, 2021) but initiatives of the European Union to reduce its dependency on imported soybean have renewed interest in lupin cultivation and the EU is currently producing 39% of the global production. Lupin anthracnose, however, is significantly hampering a further increase in lupin cultivation. As no effective sustainable treatment is available yet (Alkemade, Arncken, et al., 2022), host resistance would be the most desired solution. Recent studies identified three resistance genes in blue lupin (Fischer et al., 2015; Yang et al., 2004, 2008) and one candidate gene and two major quantitative trait loci in white lupin (Alkemade, Nazzicari, et al., 2022; Książkiewicz et al., 2017). To aid resistance breeding in lupin crops, a better understanding of *C. lupini* population structure and evolution is required as different populations may vary in geographic distribution and differ in pathogenicity, virulence, or other biological traits.

Characterization of a global *C. lupini* collection by combining isolate morphology and multilocus sequencing of four loci could distinguish six groups (I–VI; Alkemade, Messmer, Voegele, et al., 2021). However, morphological characterization has often been shown inconsistent within *Colletotrichum* (Cannon et al., 2000) and phylogeny based on four loci only gives limited information. To get insight in population structure and evolution, population genetics has been performed on a global collection of *C. lupini* isolates and members of the *C. acutatum* species complex. Genotyping of the population has been done through triple digest restriction site‐associated DNA sequencing (3D‐RADseq), which was shown to increase the number of markers at lower startup costs (Bayona‐Vásquez et al., 2019), providing an unparalleled resolution to study lupin's worst pathogen.

## 2 RESULTS

### 2.1 Four distinct lineages identified within *C. lupini*

**FIGURE 1:**
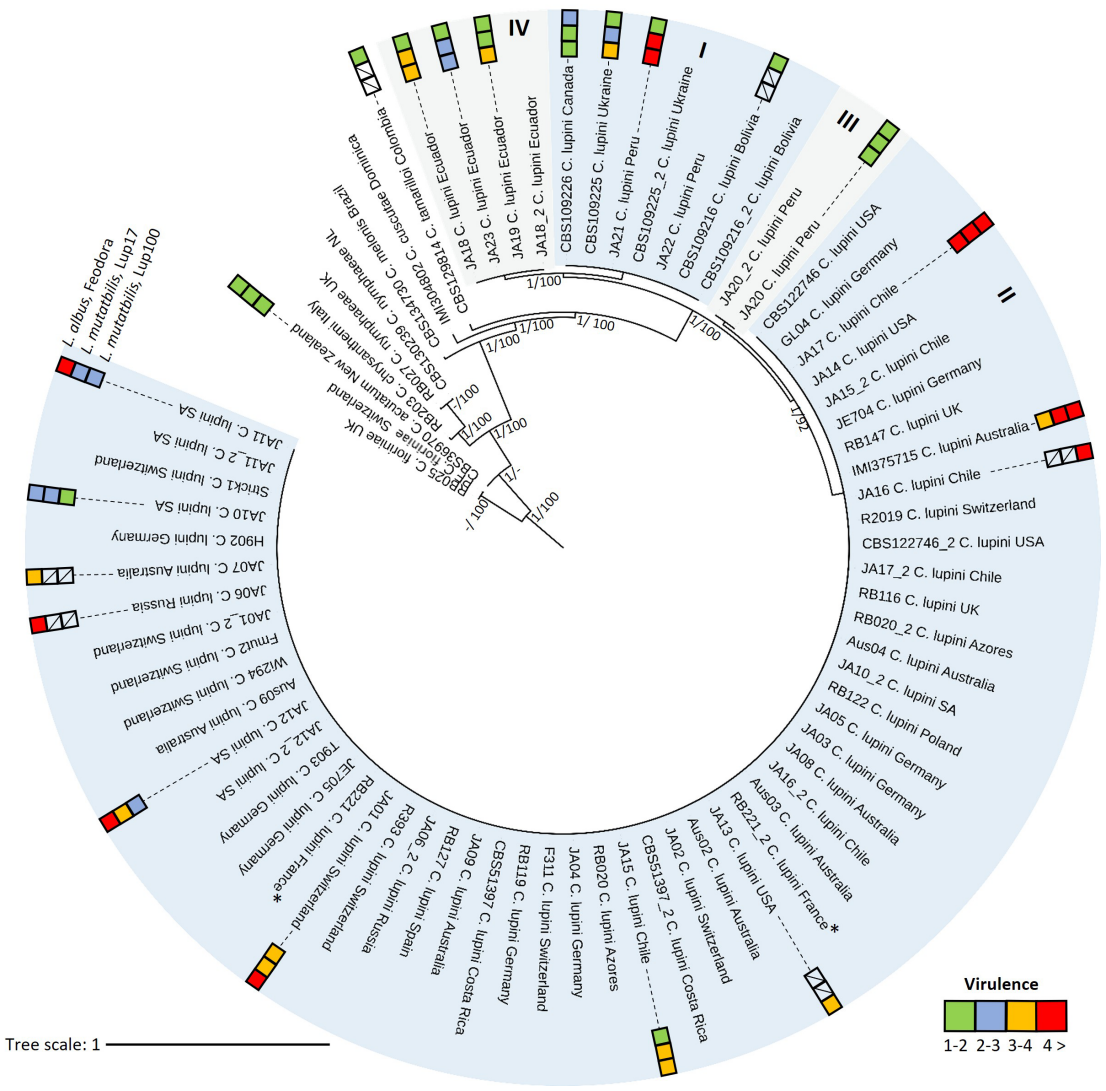
Phylogeny of *Colletotrichum lupini*. Bayesian analysis tree inferred from 9923 single‐nucleotide polymorphisms of 76 *Colletotrichum* isolates used in this study. Maximum‐likelihood bootstrap support values (>90) and Bayesian posterior probabilities (>0.95) are given at each node. The tree is rooted to *Colletotrichum fioriniae* (RB025 and PF). Isolate codes are followed by species and country of origin. For virulence (green = 1–2, blue = 2–3, orange = 3–4, and red >4) on white lupin (*Lupinus albus*) cultivar Feodora and Andean lupin (*L. mutabilis*) landraces LUP17 and LUP100, see Alkemade, Messmer, Voegele, et al. (2021) and Table S3. Isolates followed by “_2” were sequenced twice. Asterisk indicates reference genome isolate.

A total of 76 *Colletotrichum* samples (Table S1), originating from North America, South America, Europe, South Africa, and Australia, were sequenced. Genotyping through 3D‐RADseq resulted in 1,882,704 single‐nucleotide polymorphisms (SNPs) spread over 11 chromosomes after variant calling. The 16 included technical replicates showed an overall pairwise similarity of 99.4%, indicating a sequencing error rate of approximately 0.06% (Table S2). The data set was split into two data sets: one complete data set containing all sampled *Colletotrichum* species (76) and a second data set containing only *C. lupini* (67) isolates. After filtering, a total of 9923 and 1863 biallelic and phylogenetically informative SNPs spread over 10 chromosomes were retained for each data set, respectively. The mean sequencing depth was 16 before filtering and 18 after filtering the complete data set (Table S1, Figure S1). Phylogenetic analysis based on maximum likelihood and Bayesian interference was performed on the complete data set and revealed four (I–IV) well‐defined lineages within *C. lupini* (Figure 1). Within the *C. lupini* data set consisting of 1863 SNPs, 334 SNPs corresponded specifically to lineage I, 552 to lineage II, 134 to lineage III, and 147 to lineage IV (File S1). South African isolate JA10, which was previously grouped together with Peruvian isolate JA20 in group III based on multilocus sequencing and morphology (Alkemade, Messmer, Voegele, et al., 2021), firmly grouped within lineage II. Selected lineage II isolates, except for isolate JA10, showed high virulence (standardized area under the disease progress curve [sAUDPC] > 3) on white lupin, whereas selected isolates from lineage I, III, and IV showed low virulence (sAUDPC < 3) on white lupin (Figure 1; Table S3). On Andean lupin, observed virulence varies within lineages, with low and high virulence observed for selected isolates of lineage I, II, and IV. Lineage III isolate JA20 showed low virulence on both tested Andean lupin accessions. Besides differences in virulence, members of lineage I and IV also displayed a diverse intralineage morphology (Figure S2; Table S4).

### 2.2 Highest *C. lupini* diversity found in South America

**FIGURE 2:**
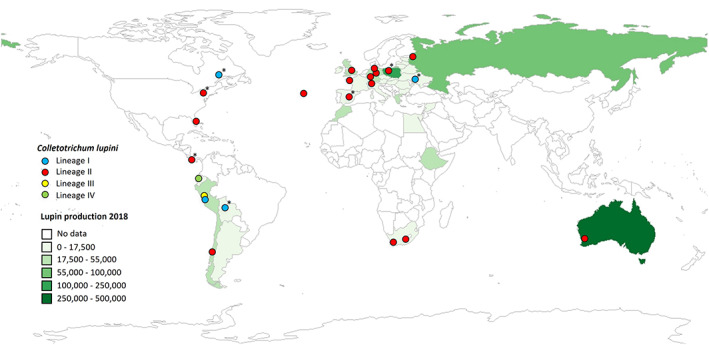
Global lupin production and distribution of *Colletotrichum lupini*. Lupin production in 2018 in tonnes. Sources are FAOSTAT (2021), Gulisano et al. (2019), and Akale et al. (2019). Blue indicates *C. lupini* lineage I, red indicates lineage II, yellow indicates lineage III, and green indicates IV. Asterisks indicate isolates collected before 1990.

**FIGURE 3:**
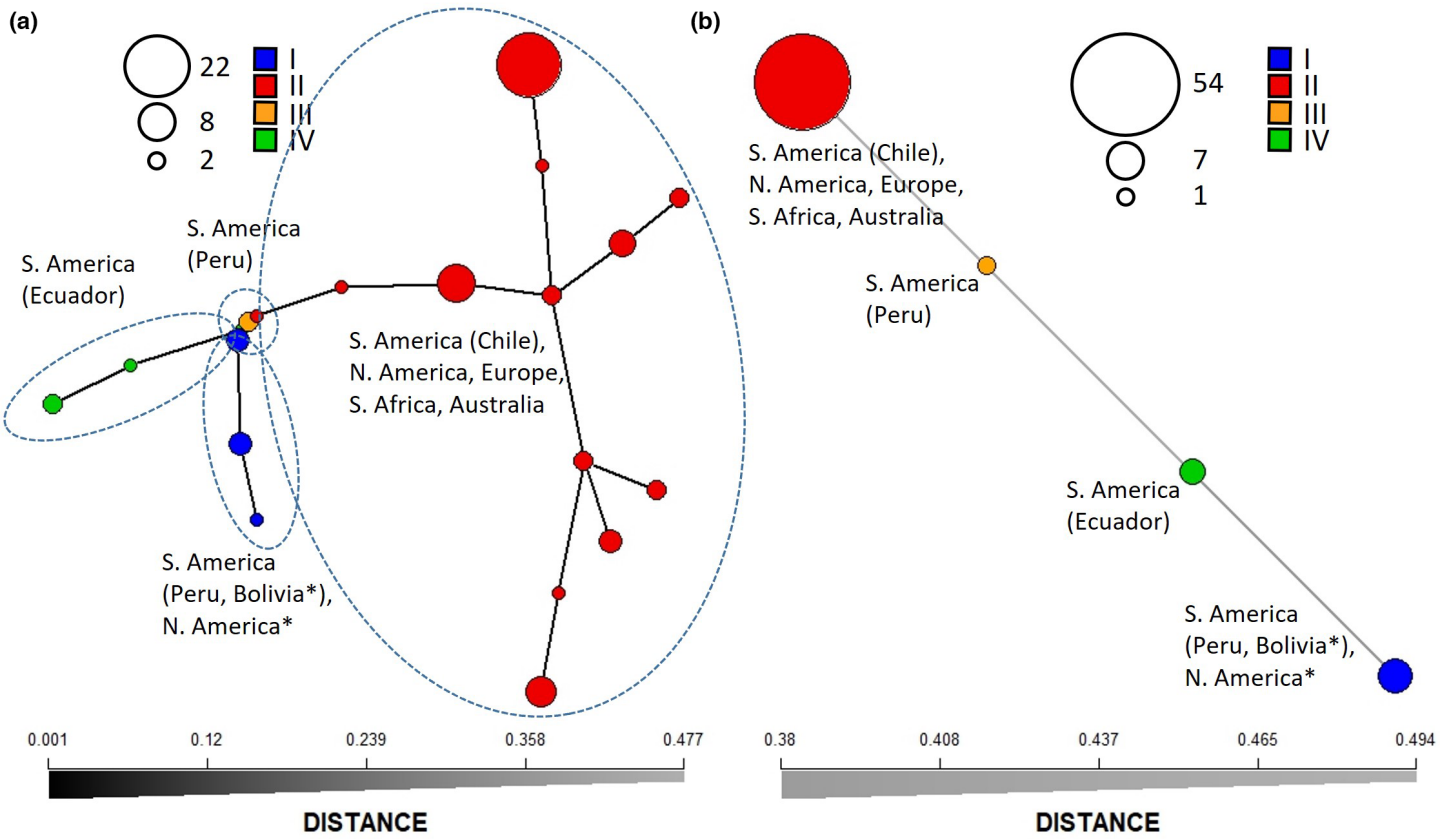
Minimum spanning networks (MSNs) showing multilocus genotypes. (a) MSN of the nonclone‐corrected *Colletotrichum lupini* data set. (b) MSN of the clone‐corrected *C. lupini* data set. Asterisk indicates regions of isolation before 1990. Analysis is based on 1863 biallelic single‐nucleotide polymorphisms.

All isolates collected outside of South America since the 1990s were grouped in lineage II, whereas isolates collected in South America were grouped across the four identified lineages (Figures 1 and 2; Table S1). The global presence of lineage II isolates and their strong virulence indicates this population to be causing the current lupin anthracnose pandemic. In South America, the four distinct lineages appeared to be geographically separated, with isolates belonging to lineage I and III being found in Peru and Bolivia, lineage II isolates being found in Chile, and lineage IV isolates being found in Ecuador (Figure 2). Separating the *C. lupini* data set based on origin (South America, North America, Europe, South Africa, and Australia) allowed for pairwise comparison between regions. Analysis of molecular variance (AMOVA) showed that most variance (70%) is explained within regions (Table 1). The generated minimum spanning networks (clone‐corrected and nonclone‐corrected) based on multilocus genotypes visualized in Figure 3 highlight that the highest diversity is found in South America. The Simpson diversity index for the nonclone‐corrected data showed Europe (0.87) and South America (0.85) to be the most diverse, compared to the other regions (0.56–0.61; Table S5). However, the Simpson diversity index for the clone‐corrected data indicated that the highest diversity was found in South America (0.72) compared to the other regions (0–0.24; Table S6). Altogether, these results contribute to the hypothesis that South America, and specifically the Andes region, is the centre of origin of *C. lupini*.

**TABLE 1:** Results of the analysis of molecular variance conducted with *Colletotrichum lupini* isolates.

	*df*	Sum of squares	Mean squares	σ	Variance component (%)	*p*
Between lineages	3	14,465	4822	644	99.86	0.001
Within lineages	63	57.18	0.91	0.91	0.14	
Total	66	14,522	220	645	100.00	
Between regions	4	4016	1004	70	29.31	0.002
Within regions	62	10,506	169	169	70.69	
Total	66	14,522	220	240	100.00	

### 2.3 Low genetic exchange between and within observed *C. lupini* lineages

**FIGURE 4:**
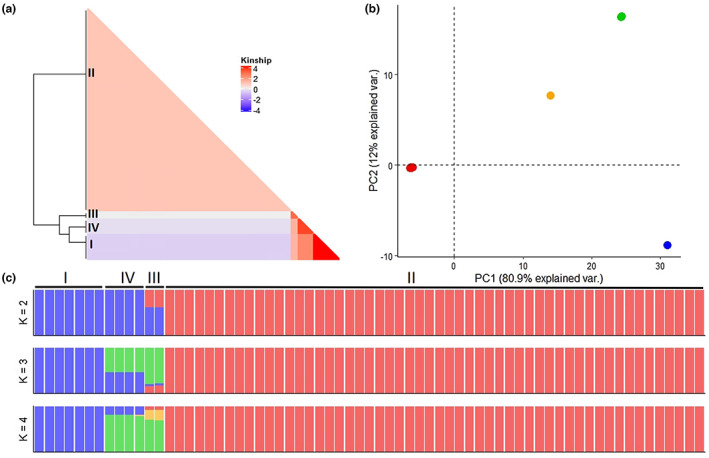
*Colletotrichum lupini* population structure. (a) Heatmap of identity‐by‐state kinship values among *C. lupini* isolates. (b) Principal component analysis. (c) Bayesian model‐based clustering analysis with different numbers of ancestral kinships (*K* = 2, 3, and 4). The *y*‐axis quantifies cluster membership and the *x*‐axis lists the different accessions. Analysis is based on 1863 biallelic single‐nucleotide polymorphisms.

We examined the genetic structure of *C. lupini* by performing Bayesian model‐based clustering analysis (Figure 4c) and found that three or four populations best matched the phylogenetic tree of Figure 1 and obtained Bayesian information criterion (BIC) and Akaike information criterion (AIC) values (Figure S3). The analysis indicated a strong population structure with low admixture between the four *C. lupini* lineages. Although interlineage exchange seems to be rare, lineage I and IV, IV and III, and III and II appear to have shared genetic information (Figure 4c) and are consequently more closely related (Figures 1 and 3). Principal component analysis (PCA), discriminant analysis of principal components (DAPC), and kinship analysis further highlighted the clear separation and low overlap between the distinct lineages (Figure 4 and Figure S4). This strong separation was also observed through AMOVA (Table 1), revealing a significant differentiation between the four different lineages (*p* = 0.001), explaining 99.88% of the observed genetic variance, whereas the observed genetic variance within lineages only accounted for 0.14%. No big difference was observed between clone‐corrected (mlg.filter: 0.05) and nonclone‐corrected (mlg.filter: 0.0008) data sets (Table 1 and Table S7). Estimates of the population differentiation statistic (*F*_ST_) further support an almost complete genetic differentiation between the lineages (Table 2), with pairwise estimates ranging from 0.81 and 0.93 between lineage II and the other lineages. Pairwise estimates between lineage I, III, and IV range from 0.20 to 0.27, indicating a weaker differentiation. Clone‐corrected data supported the presence of only four multilocus genotypes (Figure 3b), corresponding to the four observed lineages in Figure 1. An overall standardized index of association (*r̅*_d_) of 0.662 (*p* = 0.001) was found for both the clone‐corrected and nonclone‐corrected data sets (Table S8), rejecting the null hypothesis of random mating and indicating clonal reproduction. High *r̅*_d_ values were also observed for the South American, North American, and European populations, but not for the Australian and African populations. The observed results consistently show very low genetic diversity within and rare genetic exchange between the observed lineages of *C. lupini*, suggesting clonal reproduction.

**TABLE 2:** *F*_ST_ values obtained through pairwise analysis between the four *Colletotrichum lupini* lineages.

Lineage	I	II	III
II	0.81		
III	0.27	0.93	
IV	0.20	0.86	0.27

### 2.4 Variability in the presence/absence of a minichromosome

**FIGURE 5:**
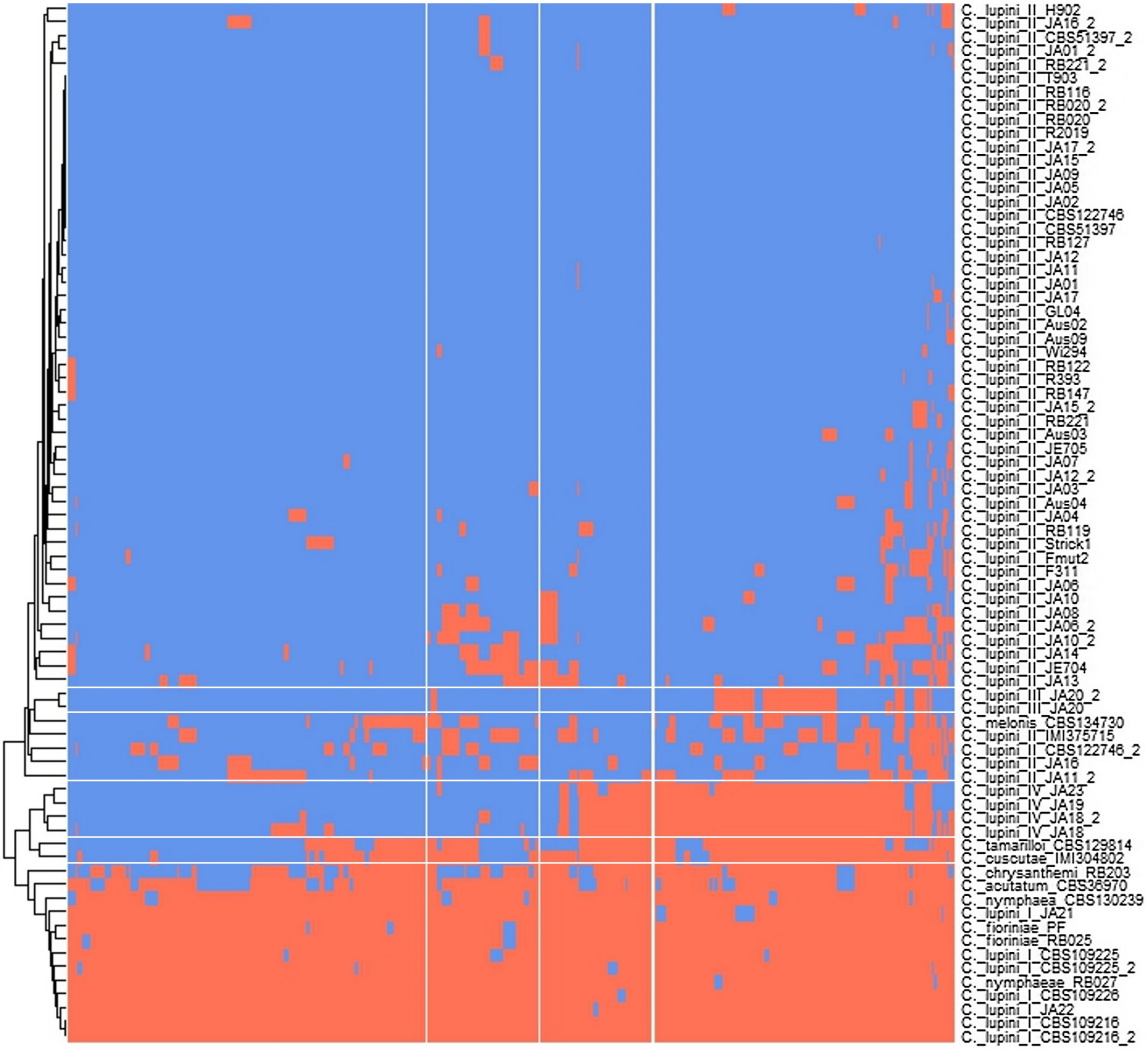
Presence/absence matrix of single‐nucleotide polymorphisms (SNPs) on minichromosome 11. A total of 5199 SNPs were called on chromosome 11 of reference genome RB221. Red indicates absence and blue indicates presence. Species are followed by lineage and strain code (see also Table S1).

A third data set was created exclusively containing markers on the 11th (mini)chromosome present in the reference genome RB211 (Baroncelli et al., 2021), by only filtering for 50% missing data, resulting in 5199 SNPs. A presence/absence matrix was constructed that showed that all members of lineage II have the minichromosome, whereas it was only partly present among members of lineage III and IV (Figure 5). Members of lineage I showed a complete absence of SNPs on minichromosome 11, comparable to *Colletotrichum* species outside of Clade 1 of the *C. acutatum* species complex. This absence was confirmed with the available genome of isolate CBS 109225 (lineage I; Baroncelli et al., 2022). A total of 57 proteins have been annotated on minichromosome 11 of the reference genome. Six of those proteins are predicted to be excreted, with three being potential effector candidates (CLUP02_18383, CLUP02_18406, and CLUP02_18404).

## 3 DISCUSSION

In this study a 3D‐RADseq approach was employed on a global collection of *Colletotrichum* isolates, resulting in a broader data set with a higher resolution compared to previous studies (Alkemade, Messmer, Voegele, et al., 2021; Dubrulle et al., 2020), with the aim to investigate the population structure and genetic diversity of the lupin pathogen *C. lupini*. Phylogenetics based on 9923 SNPs and population structure analysis based on 1863 SNPs clearly separated *C. lupini* into four independent lineages. In our previous study (Alkemade, Messmer, Voegele, et al., 2021), we suggested the presence of six distinct groups based on four loci and isolate morphology. However, classification of *Colletotrichum* and other fungal species solely based on morphological traits and a few loci can be unreliable (Cannon et al., 2000; Lardner et al., 1999). Most diversity was found in South America, in agreement with previous suggestions that South America, and specifically the Andes region, is the centre of origin of *C. lupini* (Alkemade, Messmer, Voegele, et al., 2021; Nirenberg et al., 2002; Riegel et al., 2010). This is in line with the assumption that South America is the centre of origin for members of Clade 1 of the *C. acutatum* species complex (Baroncelli et al., 2017; Bragança et al., 2016). As only a limited amount of South American isolates was analysed and many regions in South America remain unsampled, extensive sampling will possibly identify more *C. lupini* lineages.

To the best of our knowledge, only lineage I and II have escaped South America, with members of lineage I being the cause of a lupin anthracnose outbreak in the 1950s and members of lineage II causing the current pandemic (Talhinhas et al., 2016). Members of lineage I have not been found outside South America since at least 1991 and do not appear to have shared genetic information with members of lineage II in the recent past. It should be noted that different morphologies and virulence patterns were observed between the collected lineage I isolates, whereas morphologies and virulence patterns of lineage II isolates collected from all over the world were near‐identical. As a seedborne pathogen, *C. lupini* has mainly been dispersed through the trade and exchange of infected but often symptomless seeds. To prevent future outbreaks, seeds should be screened for the presence of *C. lupini* prior to exportation (Kamber et al., 2021).

The Andes region is home to a vast diversity of wild lupin species (Nevado et al., 2016) and is the area of domestication of *L. mutabilis* (Atchison et al., 2016). For Andean *Lupinus*, the estimated species diversification rate is among the fastest recorded for plants (Drummond et al., 2012). In general, the speed of plant evolution in the environmentally diverse Andes has been extraordinarily high (Hughes & Atchison, 2015; Madriñán et al., 2013). The two Andean lupin accessions screened in Alkemade, Messmer, Voegele, et al. (2021) and this study showed a diverse response to different *C. lupini* isolates representing different lineages. A collection of 10 different *L. mutabilis* accessions also showed a diverse response against a lineage II isolate (Guilengue et al., 2020). This indicates that the *L. mutabillis* gene pool harbours promising sources for crop improvement and could be used to further explore the genetic basis of host resistance. The variety of confined ecological niches and agroecosystems found across the Andes provides suitable conditions for pathogen adaptation and evolution. Together with a relatively high diversity of *C. lupini* in South America, it could be that the pathogen got engaged in a co‐evolutionary arms race with the host in the host's centre of origin. This phenomenon has been observed before for many other crop pathogens such as *F. oxysporum* on banana (Maryani et al., 2018) and *Magnaporthe oryzae* on rice (Wang et al., 2017), but not for members of the *C. acutatum* species complex (Baroncelli et al., 2017).

No sexual state has been recorded for *C. lupini* and other members of Clade 1 of the *C. acutatum* species complex (Damm et al., 2012). Vegetative compatibility groups have been described for *C. lupini* lineage I and II (Elmer et al., 2001; Shivas et al., 1998). Together with our results, showing low genetic exchange and a high overall *r̅*_d_ (0.662), this strongly indicates that at least *C. lupini* lineage II is a clonal population. However, it is likely that *C. lupini* as a species reproduces purely clonally, but due to limited samples from the presumed centre of origin South America and lineages I, III, and IV, further research is needed to confirm this. In clonal species, recombination can occur but is scarce enough to maintain a pattern of a clonal population structure (Tibayrenc & Ayala, 2012). Genetic exchange among *C. lupini* lineages has happened as admixture was observed between lineage I and IV, IV and III, and III and II. Isolates belonging to lineage I (JA21) and III (JA20) have been found in close proximity in fields near Carhuaz (Peru), which would make genetic exchange theoretically possible. Clonal populations have the ability to quickly spread as they do not need a mating partner to produce large numbers of offspring (Bazin et al., 2014; Gladieux et al., 2016). However, the complete absence of sexual recombination strongly limits adaptation potential as new genotypes can only arise through mutations or horizontal gene transfer, significantly hampering their survivability in the long term (Drenth et al., 2019). Examples of single genotypes causing significant crop losses are the broad host range *Verticillium dahliae* (Milgroom et al., 2016), *F. oxysporum* on banana (Ordonez et al., 2015), and *Phytophthora infestans* on potato (Maurice et al., 2019). Clonal populations have also been reported within *Colletotrichum*, such as *Colletotrichum kahawae* on coffee (Vieira et al., 2018) and the broad host range *Colletotrichum fioriniae* (Eaton et al., 2021). Other *Colletotrichum* species show high recombination rates, as observed for *C. graminicola* on maize (Rogério et al., 2023), *Colletotrichum truncatum* on soybean (Rogério et al., 2022), and *Colletotrichum tanaceti* on Australian pyrethrum (Lelwala et al., 2019). However, the most successful invasive fungal pathogens have both a clonal and a sexual reproductive phase (Drenth et al., 2019).

Variation was observed in the presence or absence of a minichromosome (0.5 Mb) described in the reference genome RB221, representing lineage II (Baroncelli et al., 2021). This minichromosome was present in all lineage II isolates, partly present in lineage III and IV, but completely absent in lineage I. Mini (dispensable/lineage‐specific) chromosomes tend to be highly variable, accumulating mutations and structural rearrangements more rapidly compared to the core genome, and often contain genes involved in host–pathogen interactions (Bertazzoni et al., 2018; Croll & McDonald, 2012). Transfer of minichromosomes could explain host speciation and adaptation of seemingly clonal lineages within *Fusarium* and other fungal species (Corredor‐Moreno & Saunders, 2020; Peng et al., 2019; Yang et al., 2020). For instance, transferring the minichromosome of the tomato pathogen *F. oxysporum* f. sp. *lycopersici* led to virulence of a previously nonpathogenic *Fusarium* strain (Ma et al., 2010). *Colletotrichum* minichromosomes have been reported to contribute to virulence and host speciation (Plaumann & Koch, 2020), with a minichromosome (11) found in *Colletotrichum lentis* (Bhadauria et al., 2019) and *Colletotrichum higginsianum* (Plaumann et al., 2018). In *C. lupini*, the minichromosome could play a role in virulence and host adaptation but does not seem to be required for pathogenicity on lupins for its lack in lineage I. As only three potential effector candidate genes were predicted on the minichromosome, it could also have a function unrelated to host–pathogen interaction. Further research is required to draw conclusions on its function in *C. lupini*.

Altogether, this study demonstrated the existence of four independent lineages of *C. lupini*, further validates South America to be the centre of origin, and revealed variability in the presence of a minichromosome. As the global lupin anthracnose pandemic is caused by the clonal lineage II population, breeding strategies outside of South America should focus on resistance against members of this lineage. Additionally, Andean lupin shows promise as a yet unexplored source of resistance. Disease management should focus on preventing escapes of lineages from South America. Whole‐genome sequencing could further improve our understanding of the genetic architecture of *C. lupini* related to host speciation and pathogenicity factors.

## 4 EXPERIMENTAL PROCEDURES

### 4.1 Culture collection, DNA isolation, and 3D‐RAD sequencing

*Colletotrichum* isolates were collected from public culture collections and from lupin plants with symptoms by collaborators worldwide (Table S1). A total of 51 unique *C. lupini* isolates were collected from 17 countries across five continents. Nine *Colletotrichum* species representing the genetic diversity of the *C. acutatum* species complex were also included. All isolates were single‐spored and maintained on potato dextrose agar (Carl Roth) at 22°C in the dark as working cultures. Isolates were stored in 25% glycerol at −80°C for long‐term storage. DNA of 2‐week‐old single‐spore cultures was obtained as described in Alkemade, Messmer, Voegele, et al. (2021) using a cetyltrimethylammonium bromide extraction protocol (Minas et al., 2011). The DNA concentration was adjusted to 10 ng/μL and DNA was sent to the Institute of Integrative Biology and Systems (IBIS) at Laval University (Quebec, Canada) for 3D‐RADseq library preparation. Two independent libraries with sample‐specific barcode sequences were constructed from DNA digested with restriction enzyme combinations *Pst*I/*Msp*I and *Nsi*I/*Msp*I. Libraries were combined in a single tube that was sent to Genome Quebec (Montreal, Quebec, Canada) for paired‐end (150 bp) sequencing in one lane using a TrueSeq DNA HT prep kit (Illumina, Inc.) on an Illumina Miseq instrument. A total of 16 technical replicates were included to detect technical errors.

### 4.2 SNP calling and filtering

Raw reads were inspected with FASTQC v. 0.11.9 (Babraham Bioinformatics) for quality control and demultiplexed using process radtags from Stacks v. 2.41 (Catchen et al., 2011, 2013) using default parameters. Barcodes and sequence adapters were trimmed using TRIMMOMATIC v. 0.3.8 (Bolger et al. 2014). Reads were mapped to the *C. lupini* reference genome (strain RB221, also known as IMI 504893; Baroncelli et al., 2021) using the Burrows–Wheeler aligner (BWA) v. 0.7.17 tool (Li & Durbin, 2009). The identification of SNPs was performed with FreeBayes v. 1.3.1 (Garrison & Marth, 2012) and a variant calling format (VCF) file was generated using Vcflib tools v. 1.0.1.1 (Garrison et al., 2021). SNPs were further filtered for quality (Q20), minimum sequencing depth (2), mean sequencing depth (5), minor allele count (2), minor allele frequency (0.01), and missing data (0.95) using VCFtools v. 0.1.15 (Auton & Marcketta, 2009), and only biallelic SNPs were kept.

### 4.3 Phylogenetic analysis

The SNP data were organized in two data sets. The first data set included all isolates retained after filtering. The second data set included only *C. lupini* isolates. The informloci function of R package poppr (Kamvar et al., 2014) was used in R v. 4.0.3 (R Core Team, 2020) to remove phylogenetically uninformative loci. For the phylogenetic analysis the complete data set was used. The VCF file was converted to a fasta file using the R package vcfR (Knaus & Grünwald, 2017) and multiple sequence alignment was performed with MAFFT v. 7.453 (Katoh & Standley, 2013). Phylogenetic analyses were based on maximum likelihood and Bayesian inference and were performed through the CIPRES science gateway portal (Miller et al., 2011). The maximum‐likelihood analysis was performed using RAxML v. 869 (Stamatakis, 2014) using default parameters, a GTRCAT model, and 1000 bootstrap iterations. The Bayesian inference analysis was performed using MrBayes v. 3.2.7 (Ronquist et al., 2012) using a Markov chain Monte Carlo (MCMC) algorithm using four chains and starting from a random tree topology. The analysis ran for 500,000 generations with trees sampled every 1000 generations to reach average standard deviations of split frequencies below 0.01. The first 25% of saved trees were discarded at the burn‐in phase and the 50% consensus trees and posterior probabilities were determined from the remaining trees. Bootstrap support values from the maximum‐likelihood analysis were plotted on the Bayesian phylogeny. Trees were visualized using iTOL v. 6 (Letunic & Bork, 2007).

### 4.4 Genetic differentiation and population structure

Population analysis was performed on the *C. lupini* data set. The number of multilocus genotypes was determined using the mlg.filter function by using a threshold determined by the cutoff_predictor based on Euclidean distance using poppr in R. Clone correction was performed using the mlg.filter option using a threshold of 0.05. Diversity statistics and minimum spanning networks of the clone‐corrected and nonclone‐corrected data sets were generated using the poppr and poppr.msn functions, respectively. The genetic variation among and within populations was estimated by AMOVA (Excoffier et al., 1992) using the amova function within poppr. Analysis was performed with 1000 permutations on the nonclone‐corrected and clone‐corrected data sets. Genetic differentiation among populations was assessed by calculating SNP *F*_ST_ values (Weir & Cockerham, 1984) using the genet.dist function of hierfstat (Goudet, 2005). To examine population structure, a PCA was performed using the glPca function of adegenet v. 2.1.3 (Jombart, 2008). An identity‐by‐state kinship matrix (Astle & Balding, 2009) was generated using statgenGWAS (van Rossum et al., 2020). Heatmaps were visualized using ComplexHeatmap in R. Genetic structure was further assessed by performing a nonmodel‐based DAPC using the dapc function of adegenet (Jombart, 2008). The number of retained principal components was determined using xvalDAPC and the number of clusters was identified based on BIC and AIC values. A model‐based Bayesian clustering approach was used additionally to determine admixture and clustering within *C. lupini* using STRUCTURE v. 2.3.4 (Hubisz et al., 2009). Ten independent runs for each *K* from 1 to 8 were performed with an admixture model at 2000 MCMC iterations and a 1000 burn‐in period.

### 4.5 Mode of reproduction

The index of association (*I*_A_) is a measure of multilocus linkage disequilibrium based on the variance of pairwise distances between genotypes to test the null hypothesis of random mating. The *I*_A_ and standardized index of association (*r̅*_d_), taking into account the number of loci, were calculated for both the clone‐corrected and the nonclone‐corrected data set using the ia function of poppr with 999 permutations. An *r̅*_d_ close to 0 indicates random mating, whereas an *r̅*_d_ close to 1 indicates clonal reproduction.

### 4.6 Functional analysis

Annotated protein sequences on the minichromosome (11) of the *C. lupini* reference genome (RB221) were used to predict a potential role in host interaction. We used SignalP v. 6.0 (Teufel et al., 2022) and Phobius (Käll et al., 2004) to identify secreted proteins. Candidates were excluded if they contained any transmembrane helices detected with TMHMM v. 2.0 (Krogh et al., 2001). EffectorP 3.0 (Sperschneider & Dodds, 2022) was used to identify potential effectors.

### 4.7 Virulence and morphology

Virulence tests were performed on white lupin cultivar Feodora and Andean lupin landraces LUP17 and LUP100 with *C. lupini* isolates JA01 (II), CBS19225 (I), RB121 (I), and JA23 (IV) through stem‐wound inoculation as described by (Alkemade, Messmer, Arncken, et al., 2021). The method was shown to highly correlate with three‐year field trials in Switzerland (Alkemade, Nazzicari, et al., 2022). Disease scores ranging from 1 (nonpathogenic) and 2 (low virulence) to 9 (highly virulent; Alkemade, Messmer, Arncken, et al., 2021) were taken 3, 7, and 10 days postinoculation (dpi) and the sAUDPC was calculated (Jeger & Viljanen‐Rollinson, 2001). All inoculations were performed in a growth chamber (25 ± 2°C, 16 h light, and 70% relative humidity) in a complete randomized block design with nine replicates. RB121 and JA23 morphologies were characterized as described in Alkemade, Messmer, Voegele, et al. (2021). Data were merged with available morphological and virulence data of Alkemade, Messmer, Voegele, et al. (2021). Statistical analyses were performed with R v. 4.0.3 using the packages lme4 (Bates et al., 2015), lmerTest (Kuznetsova et al., 2017), and emmeans (Lenth et al., 2020), following a mixed model with factors of interest (i.e., isolate, lupin accession) as fixed and replicated block as random factor. Data sets that did not follow assumptions of normality of residuals and homogeneity of variance were log_10_‐transformed. Data are presented as estimated least‐squares means using the aforementioned mixed model. Tukey's honestly significant difference (HSD) test (*p* ≤ 0.05) was applied for pairwise mean comparisons of the different strains within each lupin accession.

## Supporting information


**Figure S1.** Sequencing depth before filtering (a) and after filtering (b) of the complete data set.

**Figure S2.** *Colletotrichum lupini* morphology. RB121 and JA23, grouped in lineage I and IV, respectively, are from this study. Isolates indicated with an asterisk are from Alkemade, Messmer, Voegele, et al. (2021) and serve as reference for lineage associations. Strain codes are followed by country of origin and lineage (I–IV). Plates show the front and reverse of 14‐day‐old colonies on potato dextrose agar. Scale bars indicate 20 μm.

**Figure S3.** (a) Akaike information criterion (AIC) and (b) Bayesian information criterion (BIC) analysis. Indicated is the most probable number of genetic groups using the SNAPCLUST function AIC and BIC analysis.

**Figure S4.** Discriminant analysis of principal components (DAPC) and clustering of *Colletotrichum lupini* isolates. (a) Scatterplot of the DAPC using the *C. lupini* data set. DAPC was conducted with three principal components and three discriminant functions. (b) Clustering of *C. lupini* isolates. Posterior probabilities are shown on the *y*‐axis and samples are shown on the *x*‐axis. Analysis is based on 1863 biallelic single‐nucleotide polymorphisms.

**File S1.** Single‐nucleotide polymorphisms (SNPs), lineages, and chromosome positions.

**Table S1:** *Colletotrichum* isolates used in this study.

**Table S2:** Sequence similarity between technical replicates.

**Table S3:** Virulence of *Colletotrichum lupini* on white and Andean lupin. Asterisks indicate isolates screened in this study, data from other isolates come from Alkemade, Messmer, Voegele, et al. (2021). Different lowercase letters indicate significant differences between strains (Tukey HSD, *p* < 0.05).

**Table S4:** Morphology of *Colletotrichum lupini*. Asterisks indicate isolates screened in this study, data from other isolates come from Alkemade, Messmer, Voegele, et al. (2021). Different lowercase letters indicate significant differences between strains (Tukey HSD, *p* < 0.05).

**Table S5.** Diversity statistics of the nonclone‐corrected *Colletotrichum lupini* data set.

**Table S6.** Diversity statistics of the clone‐corrected *Colletotrichum lupini* data set.

**Table S7.** Results of the analysis of molecular variance conducted with the *Colletotrichum lupini* clone‐corrected data set.

**Table S8.** Linkage disequilibrium analysis within *Colletotrichum lupini* and among local populations.

## Data Availability

The data that support the findings of this study are openly available at Zenodo at https://doi.org/10.5281/zenodo.7546742.
