# Supplementary figures and images for: Attack of the clones: Population genetics reveals clonality of Colletotrichum lupini, the causal agent of lupin anthracnose
Source: Mol Plant Pathol. 2023 Apr 20;24(6):616–27. doi: 10.1111/mpp.13332 (PMC10189766; doi:10.1111/mpp.13332)

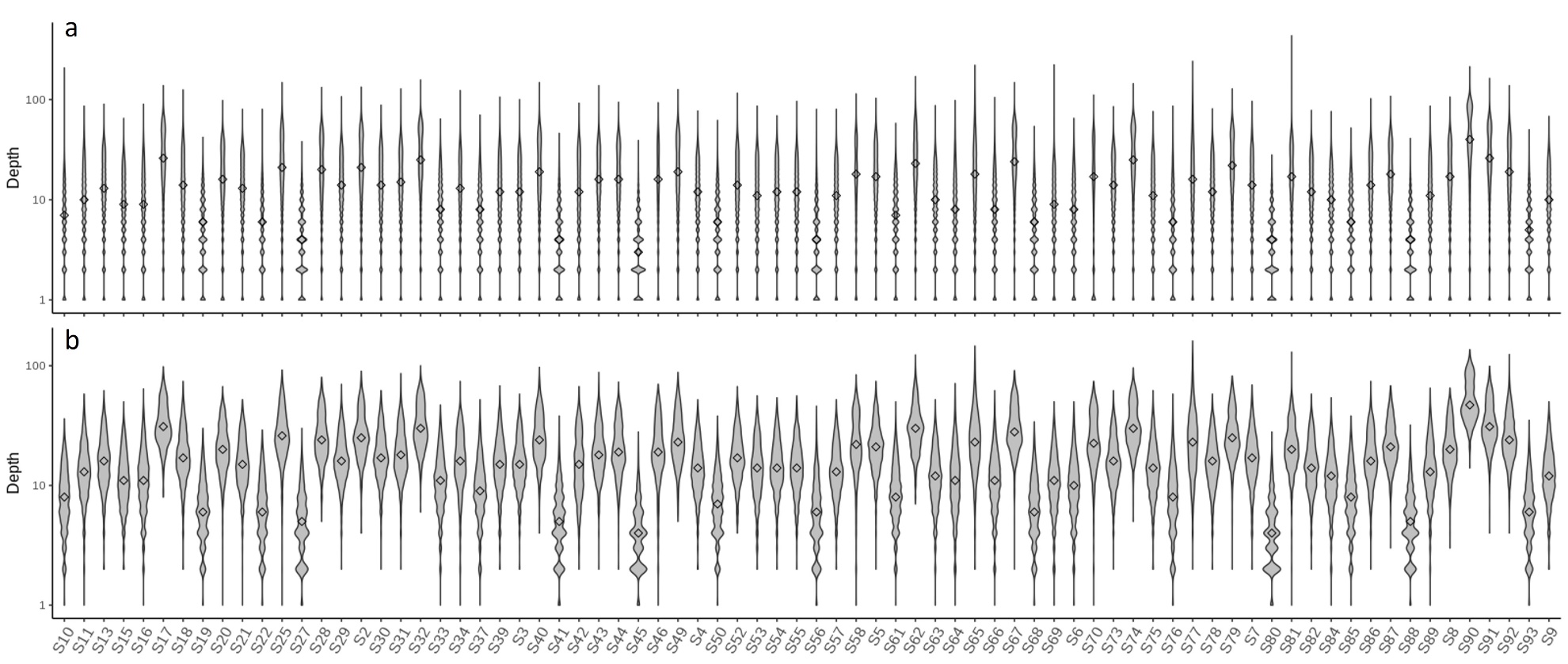


**Figure S1: Sequencing depth before filtering (a) and after filtering (b) of the complete dataset.**

Supplement: Supplementary file 1 — Figure S1. Sequencing depth before filtering (a) and after filtering (b) of the complete data set. [file MPP-24-616-s004.docx]
