# Supplementary material for: Attack of the clones: Population genetics reveals clonality of Colletotrichum lupini, the causal agent of lupin anthracnose
Source: Mol Plant Pathol. 2023 Apr 20;24(6):616–27. doi: 10.1111/mpp.13332 (PMC10189766; doi:10.1111/mpp.13332)
Supplement: Supplementary file 2 — Figure S2. Colletotrichum lupini morphology. RB121 and JA23, grouped in lineage I and IV, respectively, are from this study. Isolates indicated with an asterisk are from Alkemade, Messmer, Voegele, et al. (2021) and serve as reference for lineage associations. Strain codes are followed by country of origin and lineage (I–IV). Plates show the front and reverse of 14‐day‐old colonies on potato dextrose agar. Scale bars indicate 20 μm. [file MPP-24-616-s005.docx]

**
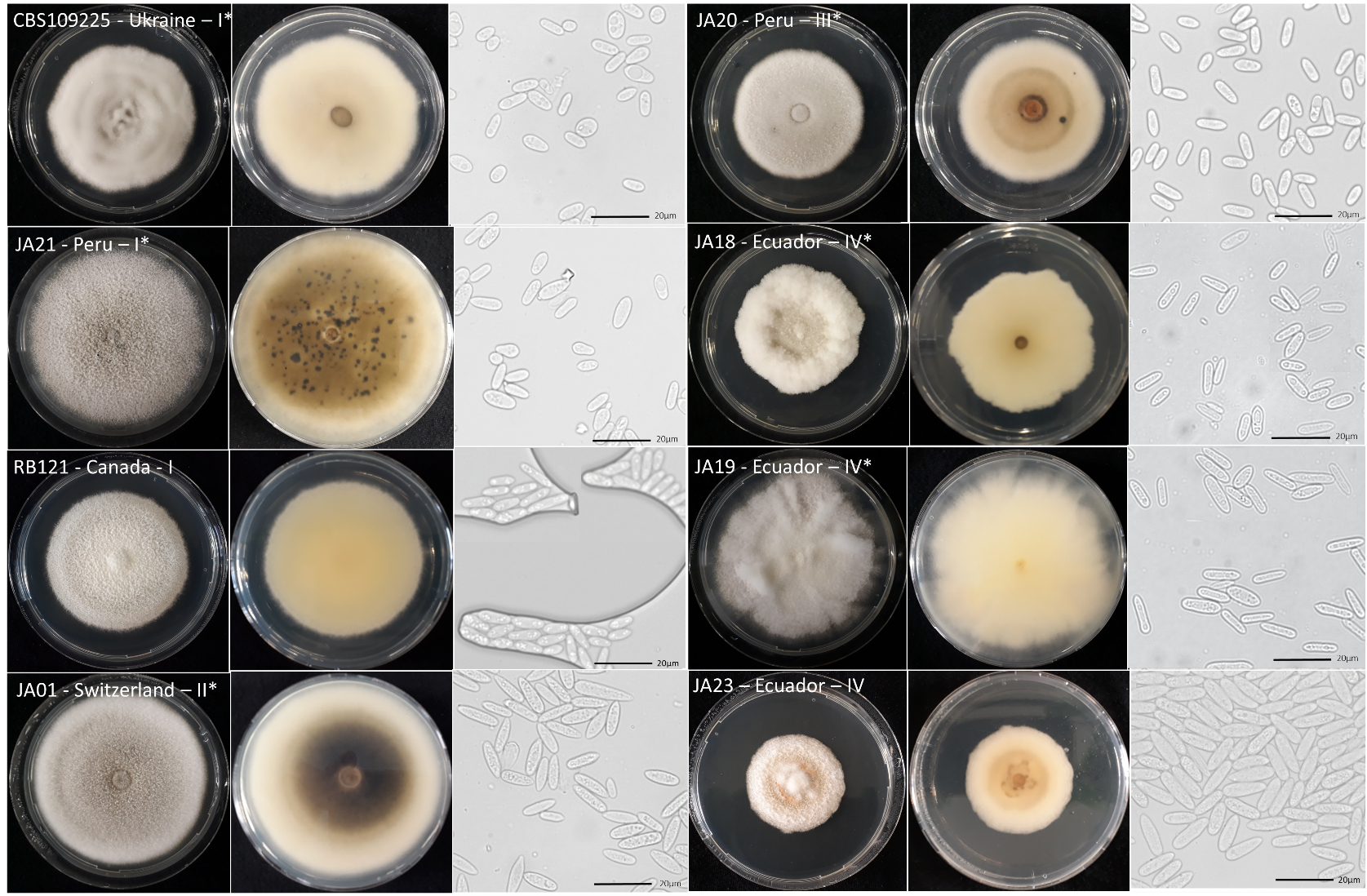
Figure S2.** ***Colletotrichum lupini* morphology.** RB121 and JA23, grouped in lineage I and IV respectively, are from this study. Isolates indicated with an asterisk are from Alkemade et al. (2021b) and serve as reference for lineage associations. Strain codes are followed by country of origin and lineage (I-IV). Plates show the front and reverse of 14 day old colonies on PDA. Scale bars indicate 20 µm.
