# Supplementary material for: Attack of the clones: Population genetics reveals clonality of Colletotrichum lupini, the causal agent of lupin anthracnose
Source: Mol Plant Pathol. 2023 Apr 20;24(6):616–27. doi: 10.1111/mpp.13332 (PMC10189766; doi:10.1111/mpp.13332)
Supplement: Supplementary file 3 — Figure S3. (a) Akaike information criterion (AIC) and (b) Bayesian information criterion (BIC) analysis. Indicated is the most probable number of genetic groups using the SNAPCLUST function AIC and BIC analysis. [file MPP-24-616-s010.docx]

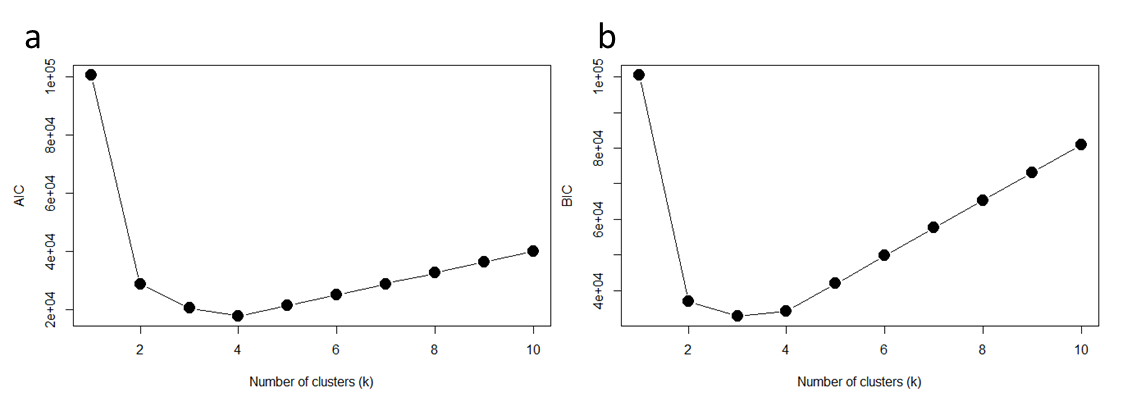


**Figure S3. (a) Akaike Information Criterion (AIC) and (b) Bayesian information criteria (BIC) analysis.** Indicating the most probable number of genetic groups using the SNAPCLUST function AIC and BIC analysis.
