# Supplementary material for: Attack of the clones: Population genetics reveals clonality of Colletotrichum lupini, the causal agent of lupin anthracnose
Source: Mol Plant Pathol. 2023 Apr 20;24(6):616–27. doi: 10.1111/mpp.13332 (PMC10189766; doi:10.1111/mpp.13332)
Supplement: Supplementary file 4 — Figure S4. Discriminant analysis of principal components (DAPC) and clustering of Colletotrichum lupini isolates. (a) Scatterplot of the DAPC using the C. lupini data set. DAPC was conducted with three principal components and three discriminant functions. (b) Clustering of C. lupini isolates. Posterior probabilities are shown on the y‐axis and samples are shown on the x‐axis. Analysis is based on 1863 biallelic single‐nucleotide polymorphisms. [file MPP-24-616-s008.docx]

**
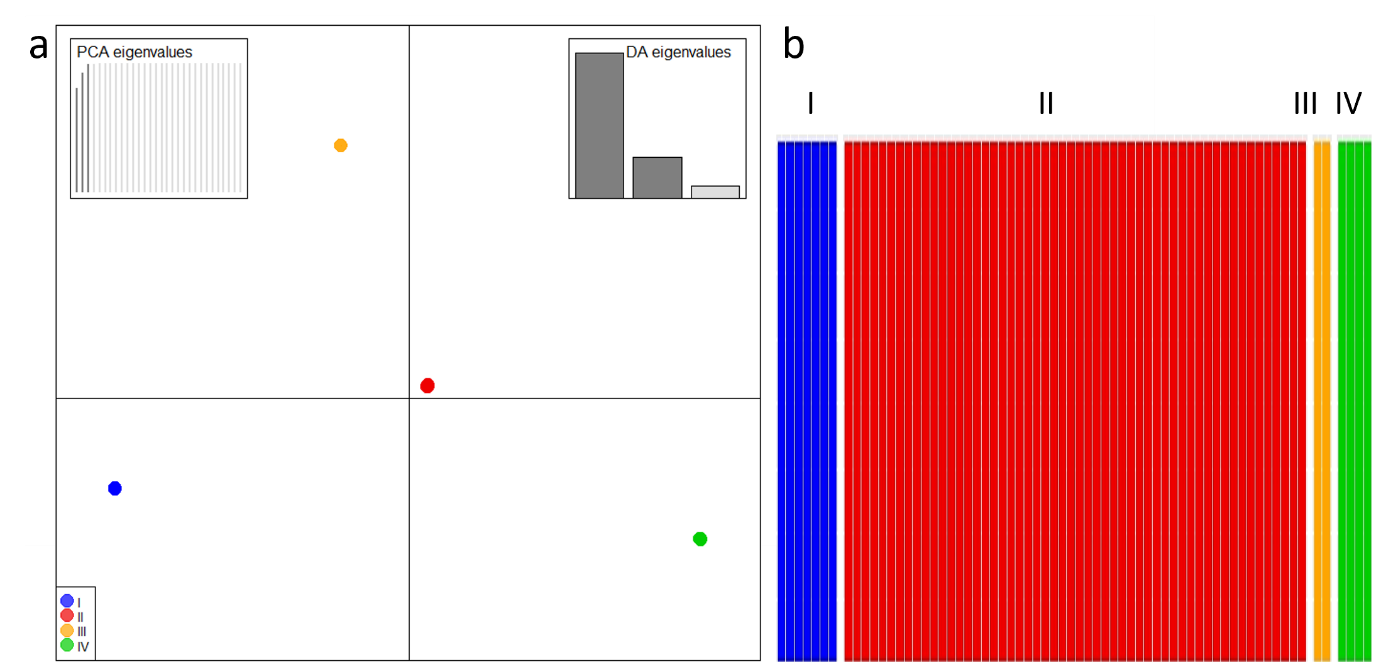
**

**Figure S4. Discriminant analysis of principal components (DAPC) and complot of *C. lupini*. (a)**, Scatterplot of the DAPC analyses using the *C. lupini* dataset. DAPC was conducted with 3 principal components and 3 discriminant functions. **(b)** Clustering of *C. lupini* isolates complot showing posterior probabilities on y axis and samples on x axis. Analysis is based on1,863 biallelic SNPs.
