# Supplementary material for: Attack of the clones: Population genetics reveals clonality of Colletotrichum lupini, the causal agent of lupin anthracnose
Source: Mol Plant Pathol. 2023 Apr 20;24(6):616–27. doi: 10.1111/mpp.13332 (PMC10189766; doi:10.1111/mpp.13332)
Supplement: Supplementary file 6 — Table S1: Colletotrichum isolates used in this study. [file MPP-24-616-s007.docx]

| **Table S1: Colletotrichum isolates used in this study**. | | | | | | | | | |
| --- | --- | --- | --- | --- | --- | --- | --- | --- | --- |
| **Isolate** | ***Colletotrichum* species** | **Host** | **Country** | **Region** | **Year** | **Alternative**  **name** | **Code** | **Mean DP BF** | **Mean**  **DP AF** |
| CBS36973 | *C. acutatum* | *Lupinus angustifolius* | New Zealand | Kumeu | - | - | S58 | 21.3 | 25.1 |
| RB203 | *C. chrysanthemi* | *Carthamus tinctorius* | Italy | Pisa | - | - | S34 | 15.9 | 18.1 |
| IMI304802 | *C. cuscutae* | *Cuscuta sp.* | Dominica | - | - | - | S93 | 6.4 | 7.0 |
| PF | *C. fioriniae* | *Malus sp.* | Switzerland | Frick | 2019 | - | S70 | 19.9 | 26.2 |
| RB025 | *C. fioriniae* | *Fragaria x ananassa* | UK | - | - | - | S46 | 19.0 | 22.7 |
| Aus02 | *C. lupini* | *Lupinus albus* | Australia | Three Springs, WA | 1996 | BRIP 63843 | S53 | 13.7 | 15.5 |
| Aus03 | *C. lupini* | *Lupinus albus* | Australia | Morawa, WA | 1996 | BRIP 63844 | S65 | 22.9 | 27.0 |
| Aus04 | *C. lupini* | *Lupinus albus* | Australia | Mingenew, WA | 1996 | BRIP 63847 | S77 | 22.0 | 27.2 |
| Aus09 | *C. lupini* | *Lupinus consentinii Guss.* | Australia | Mullewa, WA |  | 2004 | BRIP 63854 | S18 | 16.8 |
| CBS109216 | *C. lupini* | *Lupinus mutabilis* | Bolivia | - | - | - | S8 | 20.1 | 22.5 |
| CBS109216_2 | *C. lupini* | *Lupinus mutabilis* | Bolivia | - | - | - | S20 | 19.1 | 22.2 |
| CBS109225 | *C. lupini* | *Lupinus albus* | Ukraine | - | - | - | S9 | 12.4 | 14.0 |
| CBS109225_2 | *C. lupini* | *Lupinus albus* | Ukraine | - | - | - | S21 | 15.5 | 17.0 |
| CBS122746 | *C. lupini* | *Lupinus sp.* | USA | Alton, New Hampshire | - | - | S80 | 4.6 | 5.0 |
| CBS122746_2 | *C. lupini* | *Lupinus sp.* | USA | Alton, New Hampshire | - | - | S92 | 23.1 | 27.3 |
| CBS51397 | *C. lupini* | *Lupinus polyphyllus* | Costa Rica | Turialba | - | - | S32 | 29.6 | 33.6 |
| CBS51397_2 | *C. lupini* | *Lupinus polyphyllus* | Costa Rica | Turialba | - | - | S44 | 19.5 | 22.1 |
| F311 | *C. lupini* | *Lupinus albus* | Switzerland | Feldbach | 2017 | - | S37 | 9.5 | 10.7 |
| Fmut2 | *C. lupini* | *Lupinus mutabilis* | Switzerland | Feldbach | 2019 | - | S84 | 11.4 | 12.8 |
| GL04 | *C. lupini* | *Lupinus angustifolius* | Germany | Groß-Lüsewitz | 2018 | - | S86 | 16.4 | 18.9 |
| H902 | *C. lupini* | *Lupinus albus* | Germany | Hattenhofen | 2018 | - | S85 | 8.3 | 9.2 |
| IMI375715 | *C. lupini* | *Lupinus albus* | Australia | Perth, WA | 1997 | 96A4 | S45 | 4.3 | 4.6 |
| JA01 | *C. lupini* | *Lupinus albus* | Switzerland | Mellikon | 2018 | - | S52 | 16.3 | 19.1 |
| JA01_2 | *C. lupini* | *Lupinus albus* | Switzerland | Mellikon | 2018 | - | S57 | 13.1 | 14.6 |
| JA02 | *C. lupini* | *Lupinus albus* | Switzerland | Feldbach | 2019 | - | S49 | 23.2 | 26.9 |
| JA03 | *C. lupini* | *Lupinus albus* | Germany | Hattenhofen | 2019 | - | S73 | 16.0 | 17.5 |
| JA04 | *C. lupini* | *Lupinus albus* | Germany | Witzenhausen | 2018 | - | S3 | 14.7 | 16.7 |
| JA05 | *C. lupini* | *Lupinus albus* | Germany | Westerau | 2018 | - | S74 | 29.9 | 33.8 |
| JA06 | *C. lupini* | *Lupinus albus* | Russia | Saint Petersburg | 2018 | - | S11 | 12.8 | 14.9 |
| JA06_2 | *C. lupini* | *Lupinus albus* | Russia | Saint Petersburg | 2018 | - | S27 | 5.4 | 5.7 |
| JA07 | *C. lupini* | *Lupinus albus* | Australia | Dongara, WA | 2004 | BRIP 63850 | S89 | 13.6 | 15.3 |
| JA08 | *C. lupini* | *Lupinus albus* | Australia | Mingenew, WA | 2004 | BRIP 63851 | S6 | 9.9 | 11.3 |
| JA09 | *C. lupini* | *Lupinus albus* | Australia | Yan-danooka, WA | 2004 | BRIP 63857 | S30 | 17.0 | 19.5 |
| JA10 | *C. lupini* | *Lupinus albus* | SA | Bethelem | 1994 | CMW 9930, SHK 788 | S64 | 11.0 | 13.0 |
| JA10_2 | *C. lupini* | *Lupinus albus* | SA | Bethelem | 1994 | CMW 9930, SHK 788 | S74 | 13.4 | 15.4 |
| JA11 | *C. lupini* | *Lupinus albus* | SA | Stellenbosch | 1995 | CMW 9931, SHK 1033 | S5 | 19.8 | 22.8 |
| JA11_2 | *C. lupini* | *Lupinus albus* | SA | Stellenbosch | 1995 | CMW 9931, SHK 1033 | S88 | 5.3 | 5.9 |
| JA12 | *C. lupini* | *Lupinus albus* | SA | Malmsebury | 1999 | CMW 9933, SHK 2148 | S17 | 30.2 | 34.7 |
| JA12_2 | *C. lupini* | *Lupinus albus* | SA | Malmsebury | 1999 | CMW 9933, SHK 2148 | S29 | 16.2 | 18.2 |
| JA13 | *C. lupini* | *Lupinus mutabilis* | USA | Martin county, Florida | 2013 | - | S56 | 6.4 | 7.0 |
| JA14 | *C. lupini* | *Lupinus hartwegii* | USA | Martin county, Florida | 2013 | - | S68 | 7.1 | 7.6 |
| JA15 | *C. lupini* | *Lupinus albus* | Chile | Cajón | 2009 | A-02 | S54 | 14.8 | 16.0 |
| JA15_2 | *C. lupini* | *Lupinus albus* | Chile | Cajón | 2009 | A-02 | S42 | 15.5 | 17.6 |
| JA16 | *C. lupini* | *Lupinus angustifolius* | Chile | Cajón | 2009 | A-10 | S66 | 11.0 | 12.6 |
| JA16_2 | *C. lupini* | *Lupinus angustifolius* | Chile | Cajón | 2009 | A-10 | S41 | 5.7 | 6.2 |
| JA17 | *C. lupini* | *Lupinus albus* | Chile | Temuco | 2015 | A-24 | S90 | 47.6 | 54.4 |
| JA17_2 | *C. lupini* | *Lupinus albus* | Chile | Temuco | 2015 | A-24 | S78 | 15.1 | 17.2 |
| JA18 | *C. lupini* | *Lupinus mutabilis* | Ecuador | Juan Montalvo | 2007 | - | S7 | 16.4 | 19.0 |
| JA18_2 | *C. lupini* | *Lupinus mutabilis* | Ecuador | Juan Montalvo | 2007 | - | S19 | 6.8 | 7.3 |
| JA19 | *C. lupini* | *Lupinus mutabilis* | Ecuador | Pujili | 2007 | - | S43 | 18.7 | 21.1 |
| JA20 | *C. lupini* | *Lupinus mutabilis* | Peru | Carhuaz | 2019 | - | S55 | 13.8 | 15.7 |
| JA20_2 | *C. lupini* | *Lupinus mutabilis* | Peru | Carhuaz | 2019 | - | S67 | 28.8 | 32.5 |
| JA21 | *C. lupini* | *Lupinus mutabilis* | Peru | Carhuaz | 2019 | - | S79 | 25.7 | 29.0 |
| JA22 | *C. lupini* | *Lupinus mutabilis* | Peru | Carhuaz | 2019 | - | S91 | 30.6 | 35.0 |
| JA23 | *C. lupini* | *Lupinus mutabilis* | Ecuador | Cotopaxi | 2015 | - | S31 | 18.2 | 20.5 |
| JE704 | *C. lupini* | *Lupinus albus* | Germany | Jetzendorf | 2018 | - | S50 | 7.5 | 8.2 |
| JE705 | *C. lupini* | *Lupinus albus* | Germany | Jetzendorf | 2018 | - | S62 | 27.6 | 32.6 |
| R2019 | *C. lupini* | *Lupinus albus* | Switzerland | Rumikon | 2019 | - | S25 | 25.2 | 29.3 |
| R392 | *C. lupini* | *Lupinus albus* | Switzerland | Rumikon | 2017 | - | S13 | 15.9 | 17.4 |
| RB020 | *C. lupini* | *Lupinus albus* | Portugal | Azores | 1999 | PT30 | S40 | 23.5 | 27.5 |
| RB020_2 | *C. lupini* | *Lupinus albus* | Portugal | Azores | 1999 | PT30 | S82 | 14.8 | 16.5 |
| RB116 | *C. lupini* | *Lupinus polyphyllus* | UK | York | 1991 | CSL 1294 | S87 | 21.6 | 24.1 |
| RB119 | *C. lupini* | *Lupinus albus* | Germany | - | - | G52 | S33 | 10.6 | 12.2 |
| RB121 | C. lupini | Lupinus albus | Canada | - | - | CBS109226, HY09 | S16 | 11.7 | 13.0 |
| RB122 | *C. lupini* | *Lupinus luteus* | Poland | - | - | C3 | S75 | 8.1 | 9.3 |
| RB127 | *C. lupini* | *Olea europaea* | Spain | - | - | PT702 | S28 | 24.0 | 27.5 |
| RB147 | *C. lupini* | *Lupinus sp.* | UK | Kent | 1991 | IMI350308 | S4 | 14.0 | 15.3 |
| RB221 | *C. lupini* | *Lupinus albus* | France | Britanny | 2016 | IMI 504893 | S39 | 14.8 | 16.9 |
| RB221_2 | *C. lupini* | *Lupinus albus* | France | Britanny | 2016 | IMI 504893 | S63 | 11.8 | 13.0 |
| Strick1 | *C. lupini* | *Lupinus albus* | Switzerland | Strickhof | 2019 | - | S61 | 9.0 | 9.8 |
| T903 | *C. lupini* | *Lupinus albus* | Germany | Triesdorf | 2018 | - | S2 | 25.4 | 28.6 |
| Wi294 | *C. lupini* | *Lupinus albus* | Germany | Witzenhausen | 2018 | - | S15 | 11.5 | 12.5 |
| CBS134730 | *C. melonis* | *Malus sp.* | Brazil | - |  | - | - | S81 | 20.2 |
| CBS130239 | *C. nymphaeae* | *Fragaria x ananassa* | NL | - |  | - | - | S10 | 8.2 |
| RB027 | *C. nymphaeae* | *Fragaria x ananassa* | UK | Cambridge | - | - | S22 | 6.7 | 7.3 |
| CBS129814 | *C. tamarilloi* | *Solanum betaceum* | Colombia | Gundinamarca | - | - | S69 | 11.1 | 12.2 |
| Mean DP BF: mean sequencing depth before filtering. Mean DP AF: mean sequencing depth after filtering (complete dataset). | | | | | | | | | |
