# Supplementary material for: Attack of the clones: Population genetics reveals clonality of Colletotrichum lupini, the causal agent of lupin anthracnose
Source: Mol Plant Pathol. 2023 Apr 20;24(6):616–27. doi: 10.1111/mpp.13332 (PMC10189766; doi:10.1111/mpp.13332)
Supplement: Supplementary file 7 — Table S2: Sequence similarity between technical replicates. [file MPP-24-616-s013.docx]

**Table S2: Sequence similarity between technical replicates.**

| Name | Seq code | Similarity |
| --- | --- | --- |
| CBS109216 | S8 |  |
| CBS109216_2 | S20 | 99.99% |
| CBS109225 | S9 |  |
| CBS109225_2 | S21 | 99.97% |
| CBS51397 | S32 |  |
| CBS51397_2 | S44 | 100% |
| JA01 | S52 |  |
| JA01_2 | S57 | 99.99% |
| JA06 | S11 |  |
| JA06_2 | S27 | 97.40% |
| JA10 | S64 |  |
| JA10_2 | S76 | 99% |
| JA11 | S5 |  |
| JA11_2 | S88 | 97.67% |
| JA12 | S17 |  |
| JA12_2 | S29 | 100% |
| JA15 | S42 |  |
| JA15_2 | S54 | 99.99% |
| JA16 | S41 |  |
| JA16_2 | S66 | 97.23% |
| JA17 | S78 |  |
| JA17_2 | S90 | 99.96% |
| JA18 | S7 |  |
| JA18_2 | S19 | 99.31% |
| JA19 | S31 |  |
| JA19_2 | S43 | 99.98% |
| JA20 | S55 |  |
| JA20_2 | S67 | 99.99% |
| RB020 | S40 |  |
| RB020_2 | S82 | 99.98% |
| RB221 | S39 |  |
| RB221_2 | S63 | 99.81% |
| Total mean |  | 99.39% |
