# Supplementary material for: Attack of the clones: Population genetics reveals clonality of Colletotrichum lupini, the causal agent of lupin anthracnose
Source: Mol Plant Pathol. 2023 Apr 20;24(6):616–27. doi: 10.1111/mpp.13332 (PMC10189766; doi:10.1111/mpp.13332)
Supplement: Supplementary file 9 — Table S4: Morphology of Colletotrichum lupini. Asterisks indicate isolates screened in this study, data from other isolates come from Alkemade, Messmer, Voegele, et al. (2021). Different lowercase letters indicate significant differences between strains (Tukey HSD, p < 0.05). [file MPP-24-616-s006.docx]

**Table S4: Morphology of *C. lupini*.** Asterisks indicate isolates screened in this study, data from other isolates comes from Alkemade et al. (2021b). Capital letters indicate significant differences between strains (Tukey-HSD, p < 0.05).

|  | **Spore length** | | | **Spore diameter** | | | **Growth rate** | | |
| --- | --- | --- | --- | --- | --- | --- | --- | --- | --- |
| **Strain** | **Mean** | **SE** | **Tukey** | **Mean** | **SE** | **Tukey** | **Mean** | **SE** | **Tukey** |
| CBS109225-UKR-I* | 8.54 | 0.25 | a | 3.76 | 0.12 | dfg | 5.18 | 0.07 | def |
| RB121-CAN-I* | 9.55 | 0.23 | ab | 3.18 | 0.08 | bc | 4.74 | 0.07 | cd |
| CBS109216-BOL-I | 8.32 | 0.26 | a | 3.48 | 0.11 | cde | 5.59 | 0.07 | fgh |
| JA21-PER-I | 8.61 | 0.34 | a | 3.59 | 0.10 | cdf | 5.31 | 0.24 | def |
| IMI375715-AUS-II | 11.85 | 0.21 | cd | 3.90 | 0.10 | efg | 6.03 | 0.17 | gi |
| JA01-CHE-II* | 12.36 | 0.22 | ce | 4.13 | 0.08 | gh | 6.19 | 0.02 | i |
| JA06-RUS-II | 11.83 | 0.38 | c | 3.99 | 0.10 | fg | 6.21 | 0.03 | i |
| JA07-AUS-II | 11.93 | 0.35 | cd | 4.01 | 0.10 | fh | 6.13 | 0.07 | hi |
| JA10-ZAF-II | 9.16 | 0.24 | ab | 3.56 | 0.10 | cdf | 4.92 | 0.15 | de |
| JA11-ZAF-II | 12.03 | 0.23 | ce | 4.49 | 0.10 | h | 5.46 | 0.08 | eg |
| JA12-ZAF-II | 13.32 | 0.19 | e | 3.37 | 0.07 | bd | 5.62 | 0.08 | fgh |
| JA13-USA-II | 11.56 | 0.29 | c | 3.92 | 0.12 | efg | 6.08 | 0.12 | hi |
| JA15-CHL-II | 9.42 | 0.33 | ab | 4.23 | 0.15 | gh | 4.97 | 0.03 | de |
| JA16-CHL-II | 12.37 | 0.26 | ce | 3.82 | 0.11 | dfg | 6.21 | 0.05 | i |
| JA17-CHL-II | 11.96 | 0.32 | cd | 4.19 | 0.08 | gh | 6.23 | 0.04 | i |
| JA20-PER-III | 8.68 | 0.16 | a | 3.16 | 0.09 | bc | 4.18 | 0.18 | bc |
| JA18-ECU-IV | 10.03 | 0.26 | b | 2.94 | 0.10 | b | 4.08 | 0.00 | b |
| JA19-ECU-IV | 12.12 | 0.26 | ce | 2.41 | 0.10 | a | 5.97 | 0.14 | gi |
| JA23-ECU-IV* | 13.21 | 0.25 | de | 3.75 | 0.09 | dfg | 3.48 | 0.22 | a |
