# Supplementary material for: Attack of the clones: Population genetics reveals clonality of Colletotrichum lupini, the causal agent of lupin anthracnose
Source: Mol Plant Pathol. 2023 Apr 20;24(6):616–27. doi: 10.1111/mpp.13332 (PMC10189766; doi:10.1111/mpp.13332)
Supplement: Supplementary file 10 — Table S5. Diversity statistics of the nonclone‐corrected Colletotrichum lupini data set. [file MPP-24-616-s009.docx]

| Table S5. Diversity stats non-clone-corrected *C. lupini* dataset | | | | | | |
| --- | --- | --- | --- | --- | --- | --- |
| Pop | **N** | **MLG** | **H** | **G** | **lambda** | **E.5** |
| I | 7 | 3 | 1.00 | 2.58 | 0.61 | 0.91 |
| II | 54 | 13 | 2.01 | 4.72 | 0.79 | 0.58 |
| III | 2 | 1 | 0 | 1 | 1 | NA |
| IV | 4 | 3 | 1.04 | 2.67 | 0.63 | 0.91 |
| S. Africa | 6 | 3 | 1.01 | 2.57 | 0.61 | 0.90 |
| Australia | 8 | 4 | 1.07 | 2.29 | 0.56 | 0.67 |
| Europe | 30 | 12 | 2.27 | 8.04 | 0.87 | 0.81 |
| N. America | 7 | 4 | 1.15 | 2.58 | 0.61 | 0.73 |
| S. America | 16 | 8 | 1.98 | 6.74 | 0.85 | 0.92 |
| N: number of individuals, MLG: multi-locus genotypes, H: Shannon-Weiner Diversity index, G: Stoddard and Taylor’s Index, lambda: Simpson's index, E.5: evenness. | | | | | | |
