# Supplementary material for: Attack of the clones: Population genetics reveals clonality of Colletotrichum lupini, the causal agent of lupin anthracnose
Source: Mol Plant Pathol. 2023 Apr 20;24(6):616–27. doi: 10.1111/mpp.13332 (PMC10189766; doi:10.1111/mpp.13332)
Supplement: Supplementary file 11 — Table S6. Diversity statistics of the clone‐corrected Colletotrichum lupini data set. [file MPP-24-616-s012.docx]

| Table S6. Diversity stats clone-corrected *C. lupini* dataset | | | | | | |
| --- | --- | --- | --- | --- | --- | --- |
| Pop | **N** | **MLG** | **H** | **G** | **lambda** | **E.5** |
| I | 7 | 1 | 1 | 0 | 1 | 0 |
| II | 54 | 1 | 1 | 0 | 1 | 0 |
| III | 2 | 1 | 1 | 0 | 1 | 0 |
| IV | 4 | 1 | 1 | 0 | 1 | 0 |
| S. Africa | 6 | 1 | 0 | 1 | 0 | NA |
| Australia | 8 | 1 | 0 | 1 | 0 | NA |
| Europe | 30 | 2 | 0.24 | 1.14 | 0.12 | 0.51 |
| N. America | 7 | 2 | 0.41 | 1.32 | 0.24 | 0.64 |
| S. America | 16 | 4 | 1.32 | 3.56 | 0.72 | 0.93 |
| N: number of individuals, MLG: multi-locus genotypes, H: Shannon-Weiner Diversity index, G: Stoddard and Taylor’s Index, lambda: Simpson's index, E.5: evenness. | | | | | | |
