# Supplementary material for: Attack of the clones: Population genetics reveals clonality of Colletotrichum lupini, the causal agent of lupin anthracnose
Source: Mol Plant Pathol. 2023 Apr 20;24(6):616–27. doi: 10.1111/mpp.13332 (PMC10189766; doi:10.1111/mpp.13332)
Supplement: Supplementary file 12 — Table S7. Results of the analysis of molecular variance conducted with the Colletotrichum lupini clone‐corrected data set. [file MPP-24-616-s011.docx]

| **Table S7. Results of the analysis of molecular variance (AMOVA) conducted with C. lupini clone-corrected dataset** | | | | | | |
| --- | --- | --- | --- | --- | --- | --- |
|  | **Df** | **Sum of squares** | **Mean squares** | **Sigma** | **Variance component (%)** | **P-value** |
| **Between lineage** | 3 | 14484 | 4828 | 645 | 100 | 0.001 |
| **Within lineages** | 63 | 0 | 0 | 0 | 0 |  |
| **Total** | 66 | 14484 | 219 | 645 | 100 |  |
| **Between region** | 4 | 4016 | 1004 | 70 | 29.39 | 0.003 |
| **Within region** | 62 | 10468 | 169 | 169 | 70.61 |  |
| **Total** | 66 | 14484 | 219 | 239 | 100 |  |
