# Supplementary material for: Attack of the clones: Population genetics reveals clonality of Colletotrichum lupini, the causal agent of lupin anthracnose
Source: Mol Plant Pathol. 2023 Apr 20;24(6):616–27. doi: 10.1111/mpp.13332 (PMC10189766; doi:10.1111/mpp.13332)
Supplement: Supplementary file 13 — Table S8. Linkage disequilibrium analysis within Colletotrichum lupini and among local populations. [file MPP-24-616-s003.docx]

| **Table S8. Linkage disequilibrium analysis within C. lupini and among local populations.** | | | | | | |
| --- | --- | --- | --- | --- | --- | --- |
|  | | **n** | **ia** | **p-value** | **rbarD** | **p-value** |
| Overall | All isolate | 67 | 1200.803 | 0.001 | 0.662 | 0.001 |
|  | Clone corrected | 46 | 1112.734 | 0.001 | 0.609 | 0.001 |
| North America | All isolate | 7 | 1450.763 | 0.001 | 0.983 | 0.001 |
|  | Clone corrected | 6 | 1445.177 | 0.001 | 0.979 | 0.001 |
| South America | All isolate | 16 | 628.123 | 0.001 | 0.339 | 0.001 |
|  | Clone corrected | 13 | 541.199 | 0.001 | 0.292 | 0.001 |
| Europe | All isolate | 30 | 1465.166 | 0.001 | 0.987 | 0.001 |
|  | Clone corrected | 17 | 1465.874 | 0.001 | 0.987 | 0.001 |
| Africa | All isolate | 6 | 0.389 | 0.178 | 0.130 | 0.178 |
|  | Clone corrected | 5 | 0.042 | 0.540 | 0.014 | 0.540 |
| Australia | All isolate | 8 | 0.723 | 0.066 | 0.364 | 0.066 |
|  | Clone corrected | 5 | 0.167 | 0.463 | 0.083 | 0.463 |
| ia: index of association, rbarD: standardized index of association. | | | | | | |
